# A Prospective Study of Causes of Illness and Death in Preterm Infants in Ethiopia: The SIP Study Protocol

**DOI:** 10.1186/s12978-018-0555-y

**Published:** 2018-06-27

**Authors:** Lulu M. Muhe, Elizabeth M. McClure, Amha Mekasha, Bogale Worku, Alemayehu Worku, Asrat Dimtse, Goitom Gebreyesus, Zemene Tigabu, Mahlet Abayneh, Netsanet Workneh, Beza Eshetu, Abayneh Girma, Mesfin Asefa, Ramon Portales, Mahlet Arayaselassie, Yirgu Gebrehiwot, Tiruzer Bekele, Mesele Bezabih, Gesit Metaferia, Mulatu Gashaw, Bewketu Abebe, Alemu Geleta, Abdulkadir Shehibo, Yohanes Hailu, Hailu Berta, Addisu Alemu, Tigist Desta, Rahel Hailu, Janna Patterson, Assaye K Nigussie, Robert L. Goldenberg

**Affiliations:** 1 College of Medical Sciences, Addis Ababa University, Addis Ababa, Ethiopia; 2 RTI International, 3040 Cornwallis Rd., Durham, NC 27709 USA; 3 Ethiopian Pediatric Society, Addis Ababa, Ethiopia; 4 University of Gondar, Gondar, Ethiopia; 5 St Paul’s Hospital Millennium Medical College, Addis Ababa, Ethiopia; 6 Jimma University, Jimma, Ethiopia; 7 Bill and Melinda Gates Foundation, Seattle, USA; 8 Columbia University, New York, USA; 9 American Academy of Pediatrics, Itasca, Illinois USA

**Keywords:** Neonatal mortality, Preterm birth, Low-middle income countries, Cause of death

## Abstract

**Background:**

With nearly 15 million annual preterm births globally, preterm birth is the most common cause of neonatal death. Forty to 60 % of neonatal deaths are directly or indirectly associated with preterm mortality. As countries aim to meet the Sustainable Development Goals to reduce neonatal mortality, significant reductions in preterm mortality are needed. This study aims to identify the common causes of preterm illness and their contribution to preterm mortality in low-resource settings. This article will describe the methods used to undertake the study.

**Methods:**

This is a prospective, multi-centre, descriptive clinical study. Socio-demographic, obstetric, and maternal factors, and clinical and laboratory findings will be documented. The major causes of preterm mortality will be identified using clinical, laboratory, imaging, and autopsy methods and use the national Ethiopian guidelines on management of preterm infants including required investigations to reach final diagnoses. The study will document the clinical and management protocols followed in these settings. The approach consists of clinical examinations and monitoring, laboratory investigations, and determination of primary and contributory causes of mortality through both clinical means and by post-mortem examinations. An independent panel of experts will validate the primary and contributory causes of mortality.

To obtain the estimated sample size of 5000 preterm births, the study will be undertaken in five hospitals in three regions of Ethiopia, which are geographically distributed across the country. All preterm infants who are either born or transferred to these hospitals will be eligible for the study. Three methods (last menstrual period, physical examination using the New Ballard Score, and ultrasound) will be used to determine gestational age.

All clinical procedures will be conducted per hospital protocol and informed consent will be taken from parents or caretakers prior to their participation in the study as well as for autopsy if the infant dies.

**Discussion:**

This study will determine the major causes of death and illness among hospitalized preterm infants in a low-resource setting. The result will inform policy makers and implementers of areas that can be prioritized in order to contribute to a significant reduction in neonatal mortality.

## Plain English summary

When babies are born early, or preterm, they may develop conditions that place them at higher risk for short-term health problems, long-term neurological complications and even death. In the United States and other high-income countries, most infants born preterm now survive, but in the poorest regions of the world, babies who are born preterm are at very high risk of death; in the first month of life up to half may die. To date, few studies have examined the specific conditions that lead to deaths among preterm babies in low-income countries. In this paper, we describe an approach that will be used for a study about the reasons why preterm babies cared for in 5 hospitals in Ethiopia die. After consent, we will collect extensive information about all preterm babies cared for in the hospital including information on the care given during pregnancy, labor and delivery as well as for the baby at the hospital. For those babies who die, additional information, including testing for infections and with permission, examination of the body after death will also be done. A group of experts will then evaluate all the information collected to determine the final cause of the baby’s death. This information will help plan future interventions to reduce deaths among preterm infants.

## Background

National estimates of preterm birth (defined as childbirth at less than 37 completed weeks) for 184 countries have been published for the year 2010 [1] showing nearly 15 million preterm births. In the Global Burden of Disease Study, 3.1% of all disability-adjusted life-years was attributed to preterm birth, similar to the burden of HIV or malaria [2]. Globally, the estimated preterm birth rate is 11.1%. Over 60% of preterm births occur in sub-Saharan Africa and South Asia (1). Ethiopia is among the top 15 countries that contribute to two-thirds of the world’s preterm babies with an estimated preterm birth rate of 14.1% [3]. In 2015, 5.9 million children under the age of five died across the globe. Of these, 44% or 2.6 million deaths occurred within the first month of life. Just over a third of these babies died as a result of prematurity-related causes [4].

Evidence from both developed and developing countries that maintain good birth registries shows that the burden of preterm birth is increasing [5–11]. Factors possibly contributing to, but not completely explaining this upward trend, include increasing rates of multiple births, greater use of assisted reproduction techniques, increases in the proportion of births among women over 34 years of age and changes in clinical practices, such as greater use of labour induction and elective Caesarean section.

Preterm birth is a major determinant of neonatal mortality and morbidity and has long-term adverse consequences for health [12]. Complications from preterm births are the leading direct cause of neonatal deaths accounting for 35% of all newborn deaths, and are also a contributing cause in an additional 40 to 60% of neonatal deaths. Mortality rates increase proportionally with decreasing gestational age or birth weight [13–16]. In Ethiopia, of the estimated 91,700 neonatal deaths in 2010, more than one-third were estimated to be due to complications of preterm birth [17].

In the 1990’s, 70% of 11.6 million under-five deaths were due to a handful of conditions, i.e., acute respiratory infections (mostly pneumonia), diarrhoea, measles, malaria, and malnutrition and often to a combination of these conditions [18, 19]. Neonatal causes were bundled as one cause. An integrated package of interventions addressing these conditions developed in 1993 [20] by the World Health Organization (WHO) contributed to the 51% reduction of mortality to 5.9 million in 2013 compared to 1993 [21]. By 2013, the major causes of neonatal mortality were further differentiated to include sepsis and asphyxia; however, prematurity was still recorded as a single cause of neonatal mortality.

At the time when little or no care was available to manage the specific causes of mortality in preterm infants, prematurity was considered as one condition in the global disease classification nomenclature. As effective interventions become available for some of the causes of prematurity-related mortality in low resource settings, there is a need to determine the distribution of the major causes of prematurity-related mortality.

In addition to determining the causes of preterm birth, a better understanding of the sequelae of events leading to deaths in preterm infants is needed. Similar to term infants, preterm infants may suffer from multiple morbidities, such as sepsis, asphyxia, respiratory distress syndrome, major congenital malformations, and metabolic disorders [22–24]. However, the proportion that each of these conditions contributes to mortality among preterm infants is not known. The WHO has developed a simplified clinical algorithm [25] to manage sepsis among young infants below 2 months of age. Even though microbiological studies for the detection of bacteria or fungi in blood, body fluids, or relevant tissues continue to be the gold standard for diagnosis of sepsis, the yield of these cultures is generally low. Blood cultures were positive in 10% of Ethiopian young infants with suspected infection in the WHO Young Infant Study [26]. The diagnosis of respiratory distress syndrome (RDS) depends on the clinical presentation of severe respiratory distress with hypoxaemia, and a reticulo-granular pattern on a chest x-ray. The challenge is to differentiate RDS from congenitally-acquired lung infections or perinatal asphyxia. Preterm infants with intraventricular haemorrhage may not manifest any signs or only show signs of anaemia, increased head circumference, respiratory distress or neurological manifestations such as seizures. The latter symptoms can also be signs of metabolic disturbances.

To address the gap in knowledge of mortality and the morbidity associated with preterm birth, we are undertaking a prospective study of cause of preterm infant death in Ethiopia, the Study of Illness in Preterm (SIP) project.

### Objectives

The primary objective of the SIP project is to determine the most common causes of illness and mortality in preterm infants admitted to hospitals in Ethiopia based on a standardized diagnostic protocol.

Secondary objectives of the study include the following:

1. To determine the proportion of positive blood and cerebral spinal fluid cultures among preterm infants with a final diagnosis of sepsis
2. To determine the proportion of radiological findings consistent with RDS
3. To determine the proportion of head ultrasound findings consistent with intraventricular haemorrhage
4. To determine maternal/obstetric risk factors that are associated with preterm birth as well as mortality in preterm infants
5. To classify preterm infants based on gestational age (GA) and birth weight (if known) as extremely preterm (< 28 weeks) very preterm (28–32 weeks) preterm (32–37 weeks) in relation to the cause of death, to understand the GA where most of the deaths are concentrated and the corresponding birth weight if known
6. To compare the clinical final diagnosis of preterm infants that die with the results from the diagnosis obtained by standard autopsy and Minimally Invasive Tissue Sampling (MITS)

## Methods/design

### Study design

The study is a prospective multi-centre descriptive clinical study. Socio-demographic, obstetric, and maternal factors will be documented in addition to various methods of estimating gestational age before birth and at or around birth. Detailed clinical variables as well as laboratory and post-mortem findings will be recorded in a standardized manner. The major causes of preterm mortality will be identified using clinical, laboratory, imaging and autopsy methods.

Prior to the study, discussions were held with the clinical staff at each hospital regarding best practice for a number of conditions affecting the neonate, but no attempt was made to use the data collected for this study as part of a continuous quality improvement program at the study sites. In the study settings, measures such as exclusive breast feeding, keeping the baby warm, kangaroo mother care, resuscitation for perinatal asphyxia, continuous positive airway pressure (CPAP) for RDS, antibiotics for sepsis, which are routinely provided, will be provided for the study patients per clinical protocols. The study will standardize indications for investigations so that the main diagnoses can be determined. In those infants who die, attempts will be made to obtain consent from parents for a post-mortem examination.

**Fig. 1:**
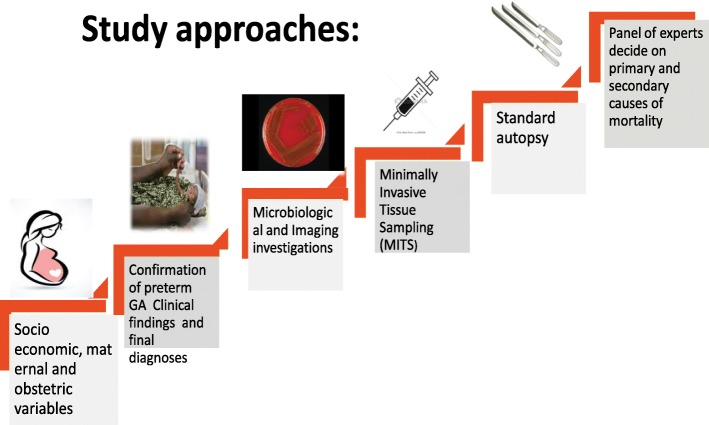
SIP Study Approaches

The study approach will consist of monitoring of clinical progress to understand the sequence of events, basic investigations and determination of primary and contributory causes of mortality followed by post-mortem examinations. Finally, an independent panel of experts will validate the primary and contributory causes of mortality (Fig. 1). The post-mortem examinations could be a whole body diagnostic autopsy or MITS method recently described in other countries [27].

The study will aim to carry out post-mortem examinations when consent is obtained from parents for autopsy. In a previous study in Addis Ababa, almost half of the bodies of the preterm infants were not collected by parents and this practice may facilitate obtaining consent for autopsy [28].

### Study settings

#### Selection of study sites

The study will be conducted in 3 locations in Ethiopia, with an intent to obtain geographical representation from several regions across the country. The main criteria for selection of hospitals for the study were prior research experience with newborn care, and the expected number of preterm infants that could be enrolled per year. Tikur Anbessa Hospital, Ghandi Memorial Hospital and St Paul Hospital in Addis Ababa, Gondar University Hospital in the north and Jimma University Hospital in south-west of Ethiopia have significant child health research experience and the largest number of newborn intensive care unit (NICU) admissions in the country.

#### Study participants

All preterm live-born infants admitted to one of the study hospitals with a gestational age of less than 37 completed weeks will be enrolled. The study hospitals admit all preterm infants that are alive without any lower limit.

Three methods (last menstrual period [LMP], physical examination using the Ballard Score, and ultrasound) will be applied for assessment of gestational age. Ultrasound (US)-predicted GA is determined by the fetal crown-rump length if taken in the first trimester and by a combination of fetal head circumference, abdominal circumference and femur length for exams performed early in the second trimester. Data on the US examination (if available) will be collected during admission of the preterm infant from the hospital records.

GA assessment is also assessed using the LMP. An accurate record of LMP means that the mother is sure of the exact date of the LMP and that her pregnancy is confirmed soon after a missing period; she had regular menstrual periods prior to conception; and she has not conceived immediately after stopping hormonal contraceptives.

The most widely accepted scoring system for postnatal estimation of GA is the New Ballard Score [29]. For SIPS, the Ballard examination will be done for all preterm infants at < 7 days of life by the study doctor to determine eligibility for the study. The new Ballard has two components: neuromuscular and physical maturity scoring. The accuracy of the Ballard is generally considered to be valid within a range of +/− 2 weeks.

For purposes of eligibility enrolment into the SIP study, if the difference between GA assessed by Ballard and that calculated from an accurate (LMP (as described above) is not greater than 2 weeks, the LMP GA will be assumed to be correct; and vice versa if the difference between the two measurements is greater than 2 weeks.

#### Screening and enrolment

Three scenarios are possible for patient screening and enrolment:

1. A woman who has been seen for antenatal care in the study hospital, goes into preterm labour, and the infant is born alive at the study hospital or home.
2. A woman who came in preterm labour to the study hospital from home or is referred from another health facility and delivers a live infant
3. A woman who delivered a preterm infant at home or in another hospital brings her preterm infant to NICU at < 7 days of life.

In all scenarios, the gestational age is estimated by a set of criteria as shown below. When preterm birth is confirmed and the consent is obtained, then the obstetric history and other clinical forms are completed.

#### Eligibility criteria

All preterm infants admitted to one of the study hospitals with a gestational age of less than 37 completed weeks and up to the age of 7 days of postnatal life will be enrolled. Three methods, i.e., ultrasound, LMP and physical examination using the new Ballard Score will be used to estimate gestational age and to be sure that an infant is indeed “preterm”. The following criteria will be used:

- Mother delivered at or baby transferred to one of the participating study hospitals;
- Gestational age is < 37 weeks according to the algorithm with the 3 methods;
- Live born defined as cry, breathing or movement after delivery or Apgar ≥ 1;
- Infant age is < 7 days when screened; and
- Consent given for study participation.

Any live born baby that meets the gestational age and age criteria will be enrolled in the study regardless of whether the baby dies prior to admission to the NICU or is discharged home without admission.

#### Exclusion criteria

- Delivery is a result of an induced abortion
- Gestational age cannot be reliably determined using study criteria.

### Study outcomes

The primary outcome of the study is determination of the main causes of mortality of preterm infants in a low-resource setting. Secondary outcomes include the determination of common preterm problems, microbiological profile of sepsis in preterm infants, maternal and obstetric risk factors for preterm mortality and comparison of clinical diagnosis against autopsy diagnosis, and comparison of standard autopsy diagnosis with that from MITS.

### Sample size

The study will take place in five hospitals each with an average yearly admission of nearly 500 preterm infants (with an average of 20% mortality rate).The study enrolment will continue for a period of 24 months to provide data on 4000 to 5000 preterm admissions across the study sites. Based on baseline data, 1000 preterm deaths are anticipated. Of those, we anticipate consent and completion of around 300 autopsies. The SIP sample size is calculated using the formula to determine sample size for a proportion:

$$ n={Z_{\frac{\alpha }{2}}}^2\frac{P\left(1-P\right)}{d^2} $$

Where.

n = the minimum sample size required for the study.

P = expected prevalence/proportion.

d = level of precision.

Zα/2 = the standard normal distribution percentile =1.96 for a 95% level of confidence.

The sample size assumed that 20% of preterm deaths are due to one of the likely causes of preterm mortality. With a 4% precision and 95% level of confidence, the sample size required will be 384preterm deaths with confirmed cause of mortality. Assuming an attrition of 10 to 20% for potential exclusions and refusal to consent to post-mortem examinations for about 50% of the cases, we will need between 770 and 960 preterm deaths. With a case fatality rate of preterm admissions of 20 to 30%, we estimate that we will need to enrol 4000 to 5000 preterm infants during the study period.

### Ethical approvals

Safety of enrolled children in this trial will be ensured by close monitoring and follow-up. All procedures except the post-mortem examinations will be conducted following the standard hospital protocols. A senior expert group led by the site coordinator will meet weekly and monitor the data and check study patients are managed according to the national guidelines. The study proposal and the forms will be reviewed and approved by institutional review committees for all participating facilities prior to study initiation.

### Informed consent for enrolment to the study

All clinical procedures will be conducted per hospital protocol and informed consent will be taken from caretakers prior to their participation in the SIP project. An informed consent form has been developed for caretakers to read (or be read to them), and understand the implications of the research and agree to sign before enrolment into the study.

### Informed consent for post-mortem examinations

For preterm infants who die, parents or caretakers will be asked to complete a separate consent for post-mortem examinations of the whole body or samples of relevant tissue or fluids. The post-mortem examination could be a complete diagnostic autopsy and/or MITS needle biopsy depending on the consent of caretakers. Infant caretakers will be read a detailed explanation for the post-mortem examination and then be asked to sign if they agree, once they have understood that the research clinician will do a whole body post-mortem examination or a sample of tissue for needle biopsy. Parents or caretakers are also allowed to take the information sheet to discuss among family members before they decide to consent to the post-mortem examinations.

### Monitoring

All preterm infants in the hospital NICU will be evaluated at least twice daily by a nurse and document the findings as per case report forms (CRFs). The site coordinator and the nursing coordinator will ensure that all forms are complete, samples for laboratory are taken in sufficient quantity and quality and the relevant lab, radiology or pathology personnel made aware of the samples sent.

The relevant clinical, laboratory, and autopsy data will be made available for a panel of experts to review every 6 months. The panel of experts will be made up of neonatologists or paediatricians, microbiologists, pathologists and epidemiologists both from internal and external sources.

### Follow up of surviving infants and determining final cause of deaths

For those infants who are discharged alive, follow up will be done weekly by phone and face to face meetings (at least once) until the age of 28 days. If the preterm infant dies at home, the WHO verbal autopsy tool will be used in addition to the hospital information to provide data to determine the cause(s) of death.

A panel of experts will be formed to evaluate cause of death. They will meet approximately every 6 months or whenever there are approximately 100 deaths that need to be evaluated for primary and contributory causes of death. The panel will review clinical data, maternal risk factors, microbiology and other related laboratory data, imaging data, autopsy data and verbal autopsy data.

Prior to the first panel meeting, guidelines for assigning the main and contributing cause of death will be distributed to the panel members to help ensure consistency in determining the cause of death. However, the cause of death will be based on the WHO categories and criteria for cause of neonatal death.

At the end of each meeting of the panel of experts, the results should be available for each case regarding clinical diagnosis and the primary and contributing cause of death by autopsy and in some cases verbal autopsy results. An analysis of the comparison of these results would indicate the amount of agreement vs. disagreement of the panel assignment with the autopsy, clinical diagnosis, and verbal autopsy.

### Data entry and data management

A senior bio-statistician will be responsible for the overall oversight of data management. The data management system (DMS) will be developed by Addis Ababa University and used at each of the participating study sites as described below.

### Data entry and data collection and transmission

The database will be constructed using Structured Query Language database. Code to check logic will be developed and integrated with the database to minimize errors during data entry. Internal validation checks such as skipping pattern, ranges, value labels and consistence checks will be programmed.

Data will be entered into computers using the DMS developed for this study. The DMS allows site staff to produce project reports and scheduled backups of the study database. Data entry clerks will be trained on the DMS system and will maintain the central database for the study. The data entry staff at each hospital site will be responsible for entering data from all completed hard copy forms into the DMS, which will be installed on the study computers. If data corrections are needed, study staff will enter the corrections into the DMS, which will create an automatic audit trail for future. The DMS has built-in quality checks to verify the accuracy and quality of the data entered.

Electronic data will be transferred on a weekly basis from each data management computer to the central data centre at AAU, creating a complete data repository. These records are uploaded on the central server and then merged with the existing database to add new records and update edited records.

Monthly audits and incomplete data reports will be performed by a review team consisting of at least the Site Coordinator and Data Entry staff person. Data editing and error resolution will be performed monthly. Additional data review will be performed at each hospital by the co-investigator.

### Data security and confidentiality

Protecting the confidentiality and ensuring the security of study data is a top priority. At the time of enrolment, each participant is given a unique participant identification number. All subsequent data collection will be linked only to the identification number rather than participant name.

### Data analysis plan

The primary analysis will be a combined analysis across all sites comparing proportions of causes of death among those who died, among those who died and had autopsies done and the proportion of similar diagnoses among those who survived. Simple comparison of proportions by categories of diseases or conditions will be done among all recruited cases against all preterm infants who were born in the hospitals. The denominator will be those infants identified as having a preterm birth with gestational age confirmed by ultrasound, LMP or the New Ballard. Other comparisons by diagnosis i.e. clinical versus autopsy, standard autopsy versus MITS, clinical diagnosis versus expert panel diagnosis will be done. Sensitivity, specificity and overall diagnostic accuracy of individual signs and combinations of signs will be calculated against final diagnosis among those who survived versus those who died. The latter will help develop clinical algorithms for common preterm diseases or conditions. The odds ratios of maternal and obstetric variables against death will also be calculated.

Further comparisons of the above outcomes will be made by gestational age (late preterm, moderate preterm and extreme preterm and various birth weight groups as well as maternal socio-economic, obstetric risk factors).

### Supervision and quality control mechanisms

Visits to each site will be carried out regularly by the Principal Investigator, the technical adviser and the clinical coordinator to check data quality and provide support as necessary. Where necessary, experience sharing will be arranged between sites so that the research capacity of institutions with less research experience will be strengthened.

The CRF (Table 1) were pretested at study hospitals for 2 weeks prior to finalization. All study staff were oriented to the CRFs as well as the manual of operations and procedures before the study was launched. The site coordinators were trained to review completed CRFs.

**Table 1:** Summary of SIP Case Report Forms

FORM Number and Name	Purpose	Who Completes
Form 00 – Screening Log	To document population screened	Research nurse/clinician
Form 01 – Eligibility Form	To confirm eligibility	Research nurse/clinician
Form 02 – Obstetric Form	Maternal history and obstetric status	Nurse/Obstetrician/pediatrician
Form 03A – Healthy Newborn Form	To assess preterm infants discharged without NICU admission	Nurse/pediatrician
Form 03B – NICU Admission/Clinical Form	To assess preterm infants admitted to NICU	Clinician/pediatrician
Form 04 – Study Monitoring Form	To monitor infants in NICU	Research nurse/clinician
Form 05 – Follow-up Form	To monitor kids discharged before day 28	Research nurse/clinician
Form 06 – Final Diagnosis at Day 28 or death	Clinical diagnosis at NICU discharge or death < 28 days	Pediatrician
Form 07 – Verbal Autopsy Form (WHO)	To document deaths which occur at home	Research nurse/opted experts from Public health
Form 08A – Complete Diagnostic autopsy	To document detailed findings of additional investigation	Pathologist
Form 08B – Minimally Invasive Tissue Sampling (MITS)		

The manual of operations and procedures and the CRF’s were developed by local and external experts, based on the national manual “Neonatal Intensive Care Unit Training- Management Protocol” published by the Federal Ministry of Health of Ethiopia in 2015. The manual consists of roles and responsibilities, definitions, clinical presentations and indications for investigations.

## Discussion

This study aims to identify the common causes of illness and death in preterm infants such that it is possible to develop an approach on the clinical management of common conditions or disease categories in preterm infants in low-resource settings. The availability of such data could facilitate the development of an integrated implementation package addressing these common conditions such that a significant reduction in neonatal mortality could be achieved. Designing an integrated approach may accelerate scaling up interventions within national programs addressing the major causes of preterm mortality in low-resource settings. Global, regional and national technical and implementing partners and donors could prioritize the package of interventions with potential maximum impact.

One of the challenges of this study will be to ensure that the study subjects are truly preterm infants. Three methods (LMP, ultrasound and New Ballard scores) will be applied for assessment of gestational age. In the study, we expect a small proportion of the infants to have ultrasound done during the first trimester of the pregnancy and LMP may not be reliable in all cases. However, the New Ballard will be used in all infants before 7 days postnatal age. While the national policy defines foetuses below 28 weeks of life as abortions, in this study, we have decided to include any infant born with signs of life or an Apgar score of 1 or more regardless of gestational age. We have not limited the minimum gestational age or weight of the preterm infant for admission to the NICU’s if they are normally admitted. Only infants admitted to the hospital prior to 7 days postnatal age are eligible for enrolment in the study because otherwise too little clinical information on the course of the first 7 days of life will likely be available. The research team will follow all infants admitted to the study until death or to the age of 28 days.

The principal result of the study is determination of the most common causes of mortality of preterm infants in a low-resource setting. The challenge is obviously to clearly define what a low resource setting neonatal care practice is. What we have done is to ensure that the study clinicians are knowledgeable on the diagnostic methods based on the national guidelines but that we do not interfere in the management in the study NICU’s. At the completion of the study and based on its results, we will make recommendations regarding the clinical protocols and investigations that could be practiced in these low-resource settings. In the study settings, measures such as early initiation and exclusive breast feeding, keeping the baby warm, kangaroo mother care, resuscitation for perinatal asphyxia, CPAP for RDS, and antibiotics for sepsis are intended to be provided as part of appropriate care in such settings. The extent to which these interventions are appropriately provided will be determined. Overall, this study should document which conditions could be better treated to reduce neonatal mortality associated with preterm birth.

## Abbreviations

CPAP: Continuous positive airway pressure
CRF: Case report forms
GA: Gestational age
IVH: Intraventricular haemorrhage
LMP: Last menstrual period
MITS: Minimally invasive tissue sampling
NICU: Neonatal intensive care unit
RDS: Respiratory distress syndrome
SIP: Study of Illness in Preterms
WHO: World health organization
